# Anti-Aging Potential of the Two Major Flavonoids Occurring in Asian Water Lily Using In Vitro and In Silico Molecular Modeling Assessments

**DOI:** 10.3390/antiox13050601

**Published:** 2024-05-14

**Authors:** Bodee Nutho, Duangjai Tungmunnithum

**Affiliations:** 1 Department of Pharmacology, Faculty of Science, Mahidol University, Bangkok 10400, Thailand; bodee.nut@mahidol.ac.th; 2 Department of Pharmaceutical Botany, Faculty of Pharmacy, Mahidol University, Bangkok 10400, Thailand

**Keywords:** Asian water lily, lotus lily, *Nymphaea lotus*, flavonoids, anti-aging, in vitro model, molecular modeling

## Abstract

Our previous study investigated the major flavonoids and antioxidant potential of Asian water lily (*Nymphaea lotus* L., family Nymphaeaceae) stamens and perianth extracts. Quercetin-3-*O*-rhamnoside (Que-3-Rha) and kaempferol-3-*O*-galactoside (Kae-3-Gal) were reported as the two most prominent flavonoids found in these extracts. Many flavonoids have been reported on the skin anti-aging effect that are useful for cosmeceutical/phytopharmaceutical application. However, Que-3-Rha and Kae-3-Gal occurring in this medicinal plant have not yet been evaluated for their ability to inhibit skin-aging enzymes. Therefore, this study aimed (1) to assess the enzyme inhibitory activity of Que-3-Rha and Kae-3-Gal, and (2) to conduct molecular modeling of these compounds against critical enzymes involved in skin aging such as collagenase, elastase, and tyrosinase. In vitro enzymatic assays demonstrated that both of the two most prominent flavonoids exhibited moderate to good inhibitory activity toward these enzymes. These experimental findings were supported by molecular docking analysis, which indicated that Que-3-Rha and Kae-3-Gal showed superior binding affinity to the target enzymes compared to the positive controls. Additionally, computational predictions suggested favorable skin permeability and no severe toxicity for both compounds. The results from molecular dynamic (MD) simulation revealed that all the complexes remained stable during the 200 ns MD simulation. Structural analyses and binding free energy calculations also supported the inhibitory potential of these two flavonoids against skin-aging enzymes. In conclusion, this study provides valuable insights into the anti-aging potential of the two major flavonoids occurring in this medicinal plant, paving the way for further development of cosmeceutical/phytopharmaceutical products targeting skin aging.

## 1. Introduction

**Figure 1:**
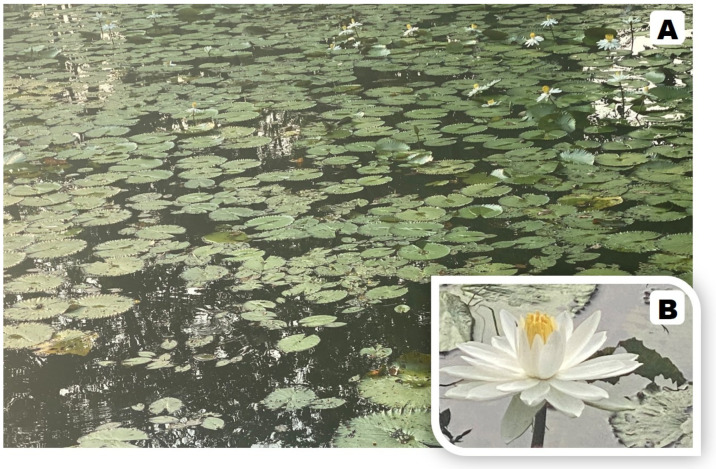
Asian water lily: (**A**) natural aquatic habitat; (**B**) flower. All photos were taken in Thailand by Duangjai Tungmunnithum.

Asian water lily, or the so-called *Nymphaea lotus* L., is a tuberous hydrophyte that grows in deep aquatic habitats of tropical/subtropical regions [1,2,3,4,5,6,7,8]. Asian water lily is a species member of Nymphaeaceae family (Figure 1). The distribution of this plant is in China, Thailand, India, Nepal, Sri Lanka, and Egypt [1,2,3,6,7,8,9,10]. In Thailand and many Asian countries, *N. lotus* is widely used as both traditional medicinal plant as well as food/vegetable. Local people in Indonesia, China, Thailand, Egypt, and other countries consume its petiole, stolon, peduncle, and perianth as a vegetable, and also use its stamen and perianth as the components of various herbal remedies and recipes [1,2,3,8,9,10,11,12,13,14].

Pharmacological activities and medicinal potentials of the extracts from Asian water lily have been investigated both in vitro and in vivo studies, e.g., anxiolytic and antidepressant effects in in vitro model [15], anti-diarrhea effect in in vivo animal model [16], and antioxidant effects in in vitro models [8,9,17,18,19]. The toxicity of this medicinal plant was also evaluated. The results from acute and sub-chronic toxicity studies on its flower extracts in an in vivo albino Wistar rat model confirmed that its flower extract exhibited antioxidant, neuroprotective activity and a boost to the body’s immune system without toxicity [15]. Furthermore, phytochemical profiles of Asian water lily have been determined, and flavonoids are reported as the potential bioactive molecules from this medicinal plant [5,9,11,18,19,20,21,22,23]. The stamen of the flower of Asian water lily was reported as the richest source flavonoids [5,8,9,11,18,19,20]. Our previous work investigated that the stamen extracts from this medicinal plant may consist of more than 475 mg/g dry weight of total flavonoids that make this species to be an interesting and valuable natural resource of flavonoid phytochemicals [19].

Additionally, there are many research studies reported on the strong correlation between the antioxidant effect of medicinal plants and their flavonoid content [12,19,22,24,25,26,27,28,29,30,31,32,33,34,35,36,37,38,39,40,41,42], especially this Asian water lily plant [8,9,12,18,19,22]. Our previous work investigated flavonoid profiles from both stamen and perianth ethanolic extracts of this medicinal plant at the population level to study the antioxidant potential of Asian water lily populations throughout Thailand using difference assays to detecting different antioxidant mechanisms [9]. Interestingly, the results confirmed that stamen consists of higher amount of flavonoids and richer antioxidant capacity comparing with that of perianth [19]. High-performance liquid chromatography (HPLC) analysis showed that quercetin-3-*O*-rhamnoside (Que-3-Rha) and kaempferol-3-*O*-galactoside (Kae-3-Gal) are the most prominent flavonoid bioactive compounds from both stamen and perianth (lower amount of flavonoid content comparing with stamen) of this medicinal plant [9]. In addition, flavonoid antioxidant compounds have been demonstrated to have various health benefits, particularly anti-aging [19,32,43]. During premature photoaging, excessive ultraviolet (UV) exposure induces the upregulation of skin-aging enzymes, especially collagenase and elastase. This upregulation leads to the breakdown of collagen and elastin, essential components for maintaining skin elasticity [44,45]. Moreover, chronic UV exposure triggers hyperpigmentation, which is associated with high activity of tyrosinase, the rate-limiting enzyme in melanin synthesis [46]. Thus, inhibiting these skin aging-related enzymes emerges as an effective strategy for skin protection and anti-aging intervention. Despite the known health benefits of flavonoids, including anti-aging properties, there is currently limited research on the anti-aging effects of Que-3-Rha and Kae-3-Gal.

Here, we report the anti-aging potential of Que-3-Rha and Kae-3-Gal against three key enzymes involved in the skin aging process, collagenase, elastase, and tyrosinase, using a combination of in vitro and in silico approaches. In vitro enzyme-based assays were initially conducted to determine the inhibitory potency of both flavonoids against these skin aging enzymes. Furthermore, multiple molecular modeling techniques were employed to gain detailed insights into the protein–ligand bindings. This approach helps in understanding their mechanism of action at the molecular level and supports the experimental results. Therefore, the results presented here provide fundamental knowledge that could pave the way for the cosmeceutical and phytopharmaceutical industries to explore alternative choices of antioxidant flavonoids that is suitable for their targeted products.

## 2. Materials and Methods

### 2.1. In Vitro Anti-Aging Activity

#### 2.1.1. Chemicals

All of the reagents were obtained from Sigma-Aldrich (St. Louis, MO, USA). All of the standard compounds (HPLC grade, Purity ≥ 98%) were purchased from Extrasynthese (Genay Cedex, France). All of the solvents used in this research study were of analytical grade, provided by Thermo Scientific (Waltham, MA, USA).

#### 2.1.2. Collagenase Assay

Collagenase of *Clostridium histolyticum* (Sigma-Aldrich) was used to conduct this assay. Collagenase activity was then examined via Shimadzu UV-2600 spectrophotometer (Shimadzu, Tokyo, Japan) by using the substrate N-[3-(2-furyl)acryloyl]-Leu-Gly-Pro-Ala (FALGPA; Sigma-Aldrich), following the protocol from Wittenauer and his team [47]. The reducing of FALGPA absorbance was then followed at 335 nm for 20 min by using the SPECTROstar ^Nano^ microplate reader (BMG labtech, Victoria, Australia). These measurements were measured in triplicate. Anti-collagenase activity was presented in the form of % inhibition relative to the control for all samples. 1,10-phenanthroline (100 μM) was employed as a specific inhibitor for this enzyme.

#### 2.1.3. Elastase Assay

An elastase assay was conducted by using porcine pancreatic elastase (Sigma-Aldrich) and the enzymatic activity was examined by Shimadzu UV-2600 spectrophotometer (Shimadzu, Tokyo, Japan) using the substrate (N-Succ-Ala-Ala-Alap-nitroanilide (AAAVPN; Sigma Aldrich)) as well as *p*-nitroaniline’s release at 410 nm using the SPECTROstar ^Nano^ microplate reader (BMG labtech, Victoria, Australia). This assay is improved from the protocol of Wittenauer and his research team [47], and these measurements were measured in triplicate. Anti-elastase potential was showed in the form of % inhibition relative to the control for all samples. Specific inhibitor of elastase was oleanolic acid (10 μM).

#### 2.1.4. Tyrosinase Assay

A tyrosinase assay was performed following the technique described by Chai and his research team [48]. In brief, the L-DOPA (5 mM; Sigma-Aldrich) was employed to be diphenolase’s substrate, then mixed in sodium phosphate buffer (50 mM, pH 6.8) and 10 μL of the samples. After that, the mushroom tyrosinase solution (Sigma Aldrich) 0.2 mg/mL was put into that mixture for a final volume 200 μL. This enzymatic reaction was measured at wavelength 475 nm using the microplate reader (BMG labtech, Victoria, Australia). Tyrosinase’s inhibitory result was reported as % inhibition relative to the control. The specific tyrosinase inhibitor was kojic acid (10 μM).

### 2.2. Computational Studies

#### 2.2.1. Ligand and Protein Structure Preparation

The SDF file formats of Que-3-Rha (PubChem CID: 5280459) and Kae-3-Gal (PubChem CID: 5282149) were downloaded from the PubChem database (https://pubchem.ncbi.nlm.nih.gov, accessed on 1 November 2023). Both compounds were then converted to the MOL2 file format using UCSF Chimera [49]. Meanwhile, known inhibitors of collagenase, elastase, and tyrosinase, including 1,10-phenanthroline (ZINC164363), oleanolic acid (ZINC3785416), and kojic acid (ZINC13831818), respectively, were retrieved from the ZINC20 database [50] (https://zinc20.docking.org/, accessed on 1 November 2023) in MOL2 file format. We employed the Gaussian09 program (Gaussian, Inc., Wallingford, CT, USA) [51] to optimize the ligands based on the B3LYP/6-31G(d) level of theory. Subsequently, the AutoDockFR 1.0 software suite [52] was utilized to convert the optimized ligands into the PDBQT file format, while the PDBQT file format of the protein *Clostridium histolyticum* collagenase G, pancreatic porcine elastase, and mushroom tyrosinase was obtained from our previous study [43].

#### 2.2.2. Molecular Docking Study

Molecular docking was performed using AutoDock Vina 1.2.5 [53]. The protein structure was considered a rigid molecule with a flexible ligand during the docking protocol. The docking site was defined based on the centroid of the co-crystallized ligand within the protein’s active site. The grid box size; grid center x, y, and z coordinates; as well as the parameter values in AutoDock Vina were set in the same manner as in our previous study [43]. The best-docked pose with the lowest AutoDock Vina docking score was selected as the initial structure for molecular dynamic (MD) simulation. Protein–ligand interactions were analyzed using UCSF Chimera [49] and ChimeraX [54] programs in 3D representation and the Discovery Studio Visualizer (BIOVIA, San Diego, CA, USA) in the 2D interaction diagrams.

#### 2.2.3. Molecular Dynamic (MD) Simulations

To gain detailed insight into the structural and dynamic behavior of Que-3-Rha and Kae-3-Gal bind in the active sites of collagenase, elastase, and tyrosinase, MD simulations were conducted using the AMBER 20 software package [55]. The initial configuration of Que-3-Rha and Kae-3-Gal complexed with each enzyme was obtained from molecular docking. The SANDER and PMEMD modules of AMBER 20 were utilized to run the MD simulations. The MD protocol was set up and performed following the same procedure as in our previous study [43]. In brief, the restrained electrostatic potential (RESP) charges of Que-3-Rha and Kae-3-Gal were obtained from the antechamber module of AMBER 20, while the general AMBER force field 2 (GAFF2) [56] was used to address missing molecular parameters of the ligands using the parmchk2 module. The TIP3P explicit water model and Na^+^ or Cl^−^ counterions were applied in each system. Following the standard protocol, each simulated protein–ligand complex underwent energy minimization, thermalization, equilibration, and was eventually transformed into the isothermal–isobaric (NPT) ensemble (1 atm and 300 K) until reaching 200 ns. Structural variations and the binding free energies of the simulated systems were calculated using the CPPTRAJ utility [57] and MMPBSA.py module [58] of AMBER 20, respectively.

#### 2.2.4. In Silico Prediction of Skin Absorption and Toxicity Assessment

The pkCSM webserver [59] (https://biosig.lab.uq.edu.au/pkcsm/, accessed on 1 November 2023) was utilized to predict logKp (skin permeation) and toxicity for Que-3-Rha and Kae-3-Gal, including AMES toxicity, hepatotoxicity, and skin sensitization. The oral toxicity (acute rodent toxicity) and toxicity classes of the compounds were predicted using the ProTox 3.0 webserver [60] (https://comptox.charite.de/protox3/, accessed on 29 March 2024).

## 3. Results and Discussion

### 3.1. In Vitro Aging-Related Enzymes Inhibition

The results from in vitro aging-related enzymes inhibition assay show that Que-3-Rha and Kae-3-Gal, which are the two most abundant major flavonoids occurring in Asian water lily medicinal plant exhibit anti-aging potential against three skin-aging enzymes (Table 1). Que-3-Rha plays an important role as an anti-aging agent for collagenase (60.24 ± 7.59% of enzyme inhibition) and elastase (50.28 ± 7.24% of enzyme inhibition), but it plays a medium role to inhibit tyrosinase (46.54 ± 6.17% of enzyme inhibition). Interestingly, Kae-3-Gal plays a key role in inhibiting collagenase (59.84 ± 8.13% of enzyme inhibition), elastase (55.56 ± 7.56% of enzyme inhibition), and tyrosinase (51.14 ± 6.89% of enzyme inhibition), respectively. The result from this current study is consistent with previously reported on the potential of flavonoids as the anti-aging phytochemical compounds for cosmetic, cosmeceutical, or phytopharmaceutical applications [5,19]. It is of note that these proteins were from non-human sources due to the commercial availability of these enzymes for in vitro testing. More importantly, collagenase from *Clostridium histolyticum* and pancreatic porcine elastase have typically been used for screening various compounds/plant extracts as inhibitors [47,61]. Similarly, tyrosinase from the mushroom, *Agaricus bisporus*, is commonly employed as an enzymatic in vitro model for developing skin-whitening substances that target human tyrosinase [48,61]. Therefore, it can be believed that the in vitro assays performed with these enzymes are reliable experiments for the initial step in evaluating anti-aging agents.

### 3.2. Molecular Docking

Molecular docking was employed to explore the binding mode and affinity of the two major flavonoid glycosides found in *N. lotus* against three crucial skin aging enzymes. The binding energies, derived from the AutoDock Vina scoring function of Que-3-Rha and Kae-3-Gal with each enzyme, are presented in Table 2. A more negative value indicates a higher binding affinity. The results revealed that both compounds exhibited lower binding energy values than those of positive controls for all target enzymes, indicating their potential to inhibit these enzymes. This observation concurs with the in vitro study (see Section 3.1), showcasing that Que-3-Rha and Kae-3-Gal could inhibit collagenase and elastase with >50% inhibition. Additionally, Kae-3-Gal demonstrated relatively good inhibition of tyrosinase, while Que-3-Rha moderately inhibited tyrosinase, associated with its lower binding affinity (less negative value) toward tyrosinase compared to Kae-3-Gal.

**Figure 2:**
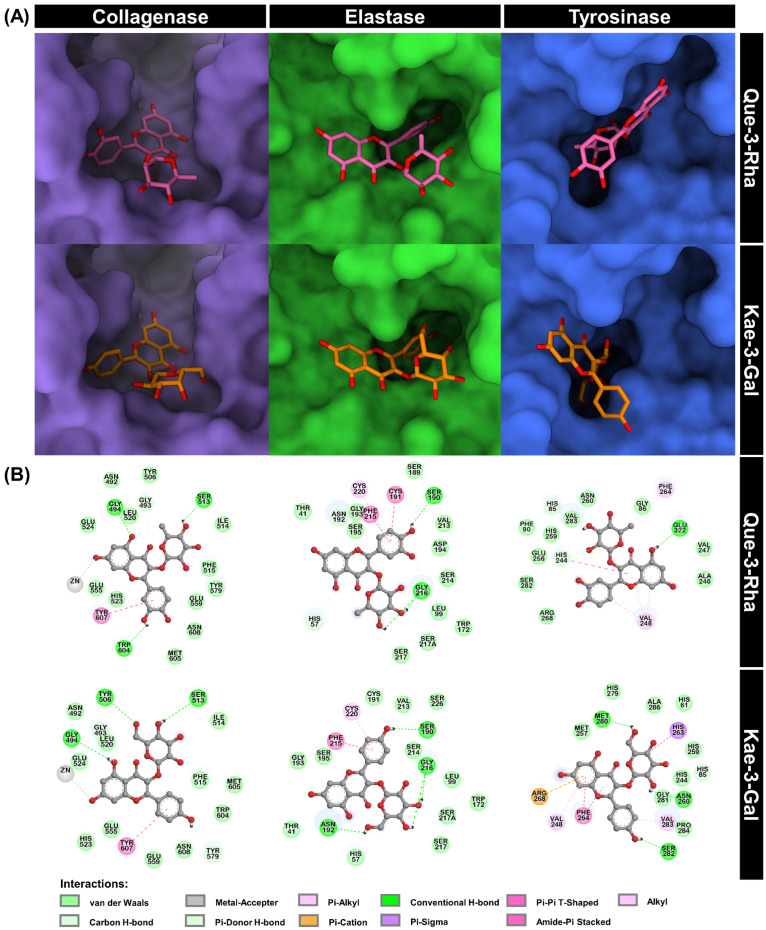
(**A**) Three-dimensional binding mode docked structures of Que-3-Rha and Kae-3-Gal bound to collagenase, elastase, and tyrosinase and (**B**) their 2D interaction diagrams.

To gain detailed insight into the protein–ligand interactions, the 3D binding mode (Figure 2A) and 2D interaction diagrams (Figure 2B) of Que-3-Rha and Kae-3-Gal bound in the active site of each target enzyme were further analyzed. The results revealed that Que-3-Rha and Kae-3-Gal effectively occupied the active site of collagenase and formed several interactions with the surrounding residues. It was observed that the hydroxyl group at the 7-position of both compounds interacted with the catalytic Zn^2+^ ion through a metal–acceptor interaction, potentially interfering with the interactions between the Zn^2+^ and its binding residues (H523, H527, and E555) during the catalytic process [62]. This observation also corroborates with our previous study of kaempferol-3-*O*-robinobioside (Kae-3-Rob), which exhibited the same type of interaction and demonstrated relatively strong inhibition toward collagenase (~60% inhibition) [43].

The docking results of elastase bound to Que-3-Rha and Kae-3-Gal showed that the two flavonoids bind to the enzyme’s active site with a highly similar orientation, particularly at the B-ring, which points toward the enzyme binding cavity. This moiety interacted with multiple residues through various interactions: (i) hydrogen bond (H-bond) with S190, amide–pi stacking with C191 and F215, and pi–alkyl with C220 for Que-3-Rha; (ii) H-bond with S190, amide–pi stacking with F215, and pi–alkyl with C220 for Kae-3-Gal. Additionally, the catalytic residues H57 and S195 formed hydrophobic contacts with both molecules, possibly leading to the inhibition of enzyme catalysis. These findings may elucidate the stronger inhibition of Que-3-Rha and Kae-3-Gal (~50–55% inhibition in Table 1) against elastase compared to our previous report of Kae-3-Rob (26% inhibition), which displayed only weak van der Waals (vdW) interaction with S195 [43]. However, a potent elastase inhibitor, epigallocatechin gallate (EGCG), could directly interact with H57 and S195 through H-bond interactions [63].

Que-3-Rha and Kae-3-Gal were also docked into the active site of tyrosinase. The results showed that the sugar moiety of both compounds was well inserted into the active site of the enzyme (Figure 2). The chromone and B-ring of the two flavonoids occupied the site close to the binding cavity entrance, potentially blocking the substrate’s accessibility to the enzyme’s active site. Furthermore, the galactose moiety of Kae-3-Gal formed a non-classical carbon H-bond with H85 and pi-sigma with H263, which are the coordinating residues of the binuclear copper ions CuA and CuB, respectively. However, the rhamnose moiety of Que-3-Rha engaged in weak vdW interactions with H85 (coordination with CuA) and H259 (coordination with CuB), which might not be strong enough to disrupt the catalytic activity of tyrosinase. This result was consistent with the slightly weaker inhibitory activity of Que-3-Rha against tyrosinase than that of Kae-3-Gal (Table 1). The disruption of the histidine-based catalytic residues was also observed in flavonoid hesperetin binding to the active site pocket of tyrosinase and interacting with three copper-coordinated residues, H61, H85, and H259 [64]. Thus, the prediction by molecular docking suggests that the inactivation of tyrosinase by Que-3-Rha and Kae-3-Gal was probably due to the binding of the copper-coordinating histidine residues in the active center of tyrosinase, thereby interfering with the redox cycle in tyrosinase catalysis.

### 3.3. Skin Permeability and Toxicity Prediction

One of the important factors in the administration of topical formulations is skin permeability, reflecting the potential for transdermal drug delivery. Therefore, we further performed in silico prediction of skin permeability, expressed as logKp (cm/h), using the pkCSM webserver. High skin permeability, defined as a logKp less than −2.5 cm/h, indicates efficient drug penetration [59]. The logKp value of Que-3-Rha and Kae-3-Gal was −2.735 (Table 3), suggesting favorable skin permeability for both compounds. Moreover, no toxicity in terms of AMES toxicity (mutagenicity), hepatotoxicity, or skin sensitization was predicted, indicating suitability for use in cosmeceutical products. The prediction of acute oral toxicity was also performed using ProTox 3.0 webserver, as summarized in Table 4. The median lethal dose (LD50) of Que-3-Rha and Kae-3-Gal was predicted at 5000 mg/kg, which belonged to toxicity class V, indicating that the compounds may be harmful if swallowed. Thus, while such theoretical predictions can guide experimental investigation of toxicity, in vitro and in vivo toxicity testing are necessary to evaluate the toxicity potential of these compounds in further studies.

### 3.4. Molecular Dynamics of Protein–Ligand Complex

#### 3.4.1. System Stability

**Figure 3:**
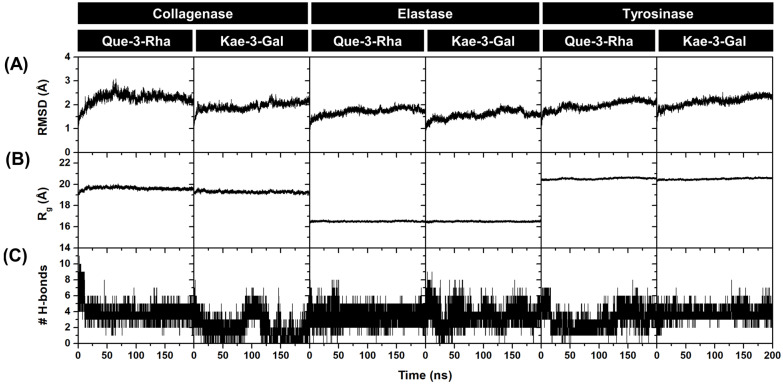
Plots of (**A**) RMSD, (**B**) R_g_, and (**C**) # H-bonds for Que-3-Rha and Kae-3-Gal in complex with collagenase, elastase, and tyrosinase during 200 ns MD simulations.

To further determine the stability and dynamic behavior of Que-3-Rha and Kae-3-Gal in complex with three aging-related enzymes in an aqueous solution, 200 ns MD simulations were conducted. Initially, root mean square deviation (RMSD) calculations of the complex atoms relative to their starting structures for each system were performed to verify the overall system stability. The time evolution of RMSDs indicated that all simulated systems reached equilibrium after approximately 100 ns (Figure 3A), showing relatively small fluctuations of approximately 1.0–2.0 Å. Moreover, we calculated the radius of gyration (R_g_) for C_α_ atoms of the protein to assess variations in the compactness of the protein structure upon ligand binding. The R_g_ profiles (Figure 3B) demonstrated that the six systems remained compact when both compounds were bound to the active site of each enzyme, with consistent R_g_ values of approximately 19.4–19.8 Å for collagenase, 16.4–16.6 Å for elastase, and 20.4–20.6 Å for tyrosinase. The number of intermolecular hydrogen bonds (# H-bonds) of enzyme binding residues with Que-3-Rha and Kae-3-Gal was monitored during the 200 ns MD simulations (Figure 3C). The time evolution of # H-bonds for the Que-3-Rha–collagenase complex displayed consistent interactions from 25 to 200 ns, with 3–4 H-bonds. Meanwhile, the # H-bond profile of Kae-3-Gal complexed with collagenase appeared to fluctuate during 75–120 ns, after which it remained constant (~1–2 H-bonds) until the end of the simulation time. The H-bond profile of elastase complexed with Que-3-Rha and Kae-3-Gal showed minor oscillation along the simulation time, especially during the 100-to-200 ns period, forming ~3–5 H-bonds. Similarly, the plot of # H-bonds for the Kae-3-Gal–tyrosinase complex revealed constant # H-bond fluctuation (3–4 H-bonds) over the course of the simulation time, whereas the tyrosinase complex of Que-3-Rha steadily formed 3–4 H-bonds from 130 to 200 ns. Taken together, our MD results, based on the structural analyses mentioned above, suggest that all the simulated systems were stable, and no significant conformational changes were observed during the 200 ns MD simulation. However, we further analyzed the MD trajectories extracted from the last 50 ns for detailed insights.

#### 3.4.2. Intermolecular Hydrogen Bonds between Protein and Ligand

**Figure 4:**
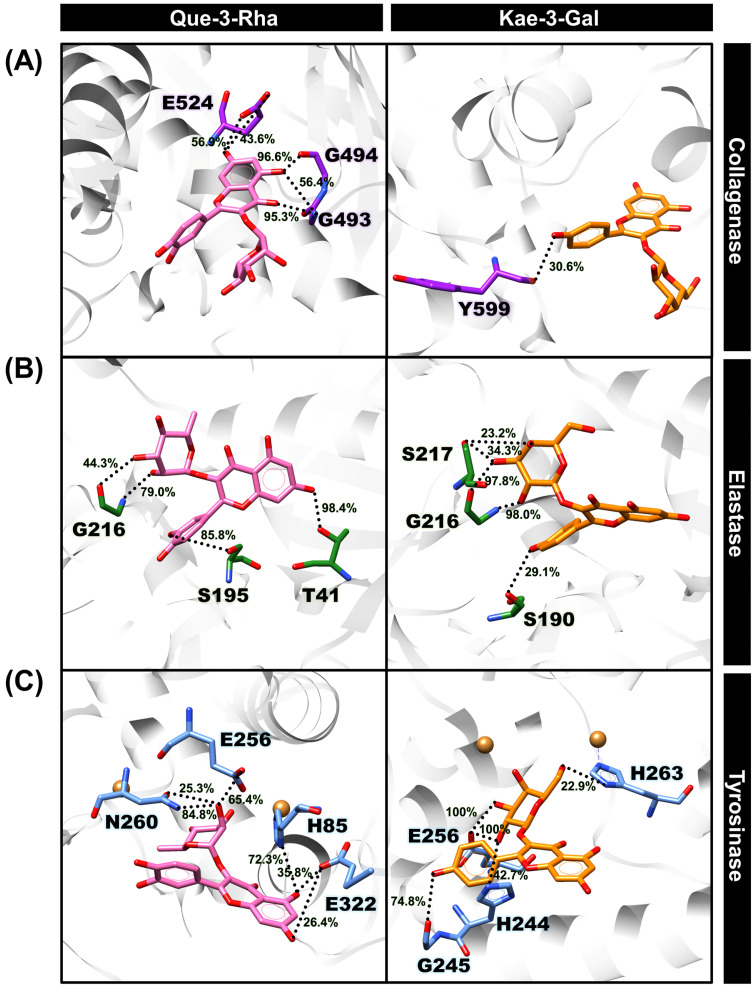
The binding patterns of Que-3-Rha (pink) and Kae-3-Gal (orange) in complex with (**A**) collagenase, (**B**) elastase, and (**C**) tyrosinase showing the percentage of H-bond occupations (black dotted lines) drawn from the last MD snapshot. Purple, green, and light blue atoms denote carbon atoms in collagenase, elastase, and tyrosinase, respectively, while red and blue atoms represent oxygen and nitrogen atoms.

As highlighted in Figure 3, H-bonds appear to play a pivotal role in the binding stabilization of Que-3-Rha and Kae-3-Gal to three skin-aging enzymes. To further evaluate this interaction, we determined the percentage of H-bond occupations between each compound and the binding residues of each enzyme during the last 50 ns simulations, based on two geometric criteria: (i) an acceptor⋯donor distance of ≤3.5 Å and (ii) an acceptor⋯H-donor angle of ≥120°. The results revealed that Que-3-Rha formed two strong H-bonds (>80% occupations) with the residue G494 (95.3% and 96.6%) to embed its chromone ring in the binding pocket of collagenase, as illustrated in Figure 4A. This finding is consistent with our previous report, demonstrating the importance of this residue in the binding of Kae-3-Rob through strong H-bonding [43]. In contrast, Kae-3-Gal exhibited only one weak H-bond (<50% occupations) with the residue Y599, which correlates with the lower number of H-bonds detected for this system (Figure 3C). Regarding the Que-3-Rha–elastase complex (Figure 4B), two strong H-bonds were found with T41 (98.4%) and S195 (85.8%), aiding in stabilizing the chromone and B-ring of Que-3-Rha, respectively. Notably, Que-3-Rha could directly form an H-bond with the catalytic residue S195, a feature not observed in the molecular docking results (Figure 2). Meanwhile, the residue G216 of elastase maintained the sugar moiety of Kae-3-Gal inside the binding pocket through two strong H-bonds (~98%). In the tyrosinase system (Figure 4C), Kae-3-Gal formed two very strong H-bonds with the residue E256 at 100%, slightly higher than the Que-3-Rha system, which formed only one strong H-bond with the residue N260 at 84.8%. This difference may be attributed to the more potent tyrosinase inhibition of Kae-3-Gal compared to Que-3-Rha, as observed in the in vitro study (Table 1).

#### 3.4.3. Binding Affinity of Protein–ligand Complex

To evaluate the binding affinities of Que-3-Rha and Kae-3-Gal to collagenase, elastase, and tyrosinase, the molecular mechanics/Poisson–Boltzmann surface area (MM/PBSA) method was employed over the set of 500 snapshots extracted during the 150–200 ns. Based on our previous study of Kae-3-Rob bound to these enzymes [43], we observed that the conformational entropic contribution (T∆S) upon ligand binding could be omitted due to the very small entropy changes in all complexes. While this end-state method may not reproduce the absolute experimental binding free energy, it has provided greater accuracy than docking scoring functions and has shown good correlation with experimental results [65,66,67]. The averaged total binding free energy ($∆G_{\mathrm{total}}$) along with its energy components of each system is presented in Table 5. Considering the molecular mechanics energy ($∆E_{\mathrm{MM}}$), the vdW interactions ($∆E_{\mathrm{vdW}}$) were the main contributor to the binding energies of the complexation of Que-3-Rha and Kae-3-Gal toward elastase, stronger than the electrostatic term ($∆E_{\mathrm{ele}}$) by approximately 2.5-fold. In contrast, the tyrosinase complexes were primarily stabilized by the $∆E_{\mathrm{ele}}$ interactions, especially for the Kae-3-Gal–elastase system, which could be attributed to the very high percentage of H-bond occupations shown in Figure 4. Whereas the $∆E_{\mathrm{vdW}}$ and $∆E_{\mathrm{ele}}$ values were closely similar for the collagenase system.

For further insight, when combining the solvation term ($∆G_{\mathrm{sol}}$), the non-polar energy ($∆E_{\mathrm{vdW}}+∆G_{\mathrm{sol}}^{\mathrm{nonpolar}}$) was considered the dominant contribution to the binding stabilization between each enzyme and the ligands, in contrast to the unfavorable energy (positive value) of the electrostatic contribution ($∆E_{\mathrm{ele}}+∆G_{\mathrm{sol}}^{\mathrm{ele}}$). Notably, the highly negative value of Kae-3-Gal in the elastase system indicated a stronger binding affinity of this compound to elastase when compared to Que-3-Rha. This finding aligns with the higher inhibitory potency observed in the in vitro enzymatic assay (Table 1). However, while the calculations may not perfectly represent the experimental results of the collagenase and tyrosinase systems, they suggest a similar binding strength of Que-3-Rha and Kae-3-Gal against tyrosinase. In addition, one of the limitations of our simulated systems may arise from the use of non-polarizable force fields (i.e., Amber FF14SB/TIP3P combination), which could impact the binding interactions of the ligands with the proteins. Therefore, MD simulations employing polarizable force fields [68] may be necessary to address this limitation in future study.

## 4. Conclusions

Our study investigated the effect of two prominent flavonoids (Que-3-Rha and Kae-3-Gal)—which are the most abundant flavonoid antioxidant bioactive compounds found in the stamen extract of the Asian water lily medicinal plant on skin-aging enzymes (collagenase, elastase, and tyrosinase)—through both in vitro and in silico evaluations. Initially, we conducted in vitro enzymatic assays to assess the inhibitory potential of these flavonoids against the target enzymes. The results revealed that Que-3-Rha exhibited significant anti-aging effects on collagenase and elastase, although it showed weaker inhibition against tyrosinase. Meanwhile, Kae-3-Gal demonstrated inhibition of collagenase, elastase, and tyrosinase by more than 50%. These findings were consistent with the scores obtained from molecular docking. In silico predictions also indicated favorable skin permeability and no severe toxicities for both compounds. Additionally, 200 ns MD simulations were carried out to explore the dynamic behavior and binding interactions of Que-3-Rha and Kae-3-Gal with each enzyme. The results indicated stable conformations of all the protein–ligand complexes throughout the simulations. Furthermore, our MD analysis displayed that H-bond formations contributed to the stabilization of the binding of Que-3-Rha and Kae-3-Gal with each enzyme, particularly in the case of the tyrosinase complex of Kae-3-Gal. The MM/PBSA-based binding free energy calculations supported the inhibitory potential of these two flavonoids against the three skin-aging enzymes. Overall, our study provides valuable insights into the anti-aging properties of two major flavonoids occurring in this medicinal plant, laying the groundwork for the development of potential skin anti-aging molecules.

Our current research showcases the anti-aging potential of Que-3-Rha and Kae-3-Gal, antioxidant bioactive compounds found in Asian water lily, as an alternative active ingredient for cosmeceutical and other phytopharmaceutical product development. For future research directions, it is imperative to determine the safety, efficacy, and stability of the developed cosmeceutical and other phytopharmaceutical products. Furthermore, studies in in vivo models and clinical trials are necessary to assess the viability of herbal drug developments in future work.

## Figures and Tables

**Table 1:** Comparison of in vitro skin aging enzyme inhibition of Que-3-Rha and Kae-3-Gal.

Anti-Aging Activities	% of Enzyme Inhibition ^a^	
	Que-3-Rha	Kae-3-Gal
Collagenase	60.24 ± 7.59	59.84 ± 8.13
Elastase	50.28 ± 7.24	55.56 ± 7.56
Tyrosinase	46.54 ± 6.17	51.14 ± 6.89

^a^ 1,10-phenanthroline (100 µM) was employed as the specific inhibitor of collagenase, leading to an inhibition of 32.1 ± 1.8%, whereas oleanolic acid (10 µM) was used as the specific inhibitor of elastase, leading to an inhibition of 45.9 ± 1.4%. Kojic acid (10 µM) was employed as the specific inhibitor of tyrosinase, leading to an inhibition of 51.1 ± 0.9%.

**Table 2:** Docking score of Que-3-Rha and Kae-3-Gal against collagenase, elastase, and tyrosinase obtained from AutoDock Vina scoring function.

Compound	Binding Energy (kcal/mol)		
	Collagenase	Elastase	Tyrosinase
Que-3-Rha	−8.231	−7.817	−6.629
Kae-3-Gal	−7.602	−7.938	−7.261
1,10-phenanthroline	−5.502	–	–
Oleanolic acid	–	−6.867	–
Kojic acid	–	–	−5.574

**Table 3:** Skin permeability and toxicity assessment of Que-3-Rha and Kae-3-Gal predicted by pkCSM webserver.

Compound	Skin Permeability (logKp) ^a^	Toxicity		
		AMES Toxicity	Hepatotoxicity	Skin Sensitization
Que-3-Rha	−2.735	No	No	No
Kae-3-Gal	−2.735	No	No	No

^a^ A compound is considered to have a relatively low skin permeability if it has a logKp > −2.5.

**Table 4:** Prediction of acute oral toxicity and toxicity class of Que-3-Rha and Kae-3-Gal predicted by ProTox 3.0 webserver.

Compound	LD50 Predicted in Rodents (mg/kg)	Toxicity Class ^a^
Que-3-Rha	5000	5
Kae-3-Gal	5000	5

^a^ Class I: fatal if swallowed (LD50 ≤ 5); Class II: fatal if swallowed (5 < LD50 ≤ 50). Class III: toxic if swallowed (50 < LD50 ≤ 300). Class IV: harmful if swallowed (300 < LD50 ≤ 2000). Class V: may be harmful if swallowed (2000 < LD50 ≤ 5000). Class VI: non-toxic (LD50 > 5000).

**Table 5:** Total binding free energy and its energetic components (kcal/mol) for the complexation between the three skin aging enzymes (collagenase, elastase, and tyrosinase) and the two flavonoids (Que-3-Rha and Kae-3-Gal).

	Collagenase		Elastase		Tyrosinase	
	Que-3-Rha	Kae-3-Gal	Que-3-Rha	Kae-3-Gal	Que-3-Rha	Kae-3-Gal
$∆E_{\mathrm{vdW}}$	−22.32 ± 0.19	−16.39 ± 0.20	−32.18 ± 0.15	−34.89 ± 0.14	−29.08 ± 0.15	−25.01 ± 0.17
$∆E_{\mathrm{ele}}$	−22.06 ± 0.14	−18.65 ± 0.27	−14.21 ± 0.15	−14.48 ± 0.16	−31.57 ± 0.27	−41.18 ± 0.17
$∆E_{\mathrm{MM}}$	−44.38 ± 0.21	−35.04 ± 0.30	−46.39 ± 0.19	−49.37 ± 0.19	−60.65 ± 0.27	−66.19 ± 0.15
$∆G_{\mathrm{sol}}^{\mathrm{ele}}$	25.82 ± 0.17	26.14 ± 0.22	27.86 ± 0.15	22.92 ± 0.12	43.52 ± 0.25	49.45 ± 0.16
$∆G_{\mathrm{sol}}^{\mathrm{nonpolar}}$	−3.21 ± 0.02	−2.98 ± 0.01	−4.02 ± 0.01	−3.60 ± 0.01	−4.04 ± 0.01	−3.56 ± 0.01
$∆G_{\mathrm{sol}}$	22.61 ± 0.16	23.16 ± 0.21	23.84 ± 0.15	19.32 ± 0.12	39.48 ± 0.24	45.89 ± 0.15
$∆G_{\mathrm{total}}$	−21.77 ± 0.13	−11.88 ± 0.16	−22.55 ± 0.14	−30.05 ± 0.12	−21.17 ± 0.16	−20.30 ± 0.14

Data are shown as mean ± standard error of the mean (SEM).

## Data Availability

All the data supporting the findings of this study are included in this article.
